# Bioactive Peptide Profiling in Collagen Hydrolysates: Comparative Analysis Using Targeted and Untargeted Liquid Chromatography–Tandem Mass Spectrometry Quantification

**DOI:** 10.3390/molecules29112592

**Published:** 2024-05-31

**Authors:** Merve Oztug

**Affiliations:** 1 TUBITAK National Metrology Institute (TUBITAK UME), Kocaeli 41470, Turkey; merve.oztug@tubitak.gov.tr; 2 Faculty of Science and Letters, Department of Molecular Biology and Genetics, Istanbul Technical University, Istanbul 34469, Turkey

**Keywords:** collagen hydrolysates, bioactive peptides, LC-MS/MS, peptide profiling, propyl chloroformate derivatization

## Abstract

The investigation of collagen hydrolysates (CHs) is essential due to their widespread use in health, cosmetic, and therapeutic industries, attributing to the presence of bioactive dipeptides (DPs) and tripeptides (TPs). This study developed a novel targeted liquid chromatography–tandem mass spectrometry (LC-MS/MS) method with propyl chloroformate (PCF) derivatization to measure three bioactive peptides—Hydroxyprolyl-glycine (Hyp-Gly), Glycyl-prolyl-hydroxyproline (Gly-Pro-Hyp), and Prolyl-hydroxyproline (Pro-Hyp)—in CHs, with strong correlation coefficients (0.992, 1.000, and 0.995, respectively) and low limits of detection (LODs) of 1.40, 0.14, and 1.16 µM, respectively. Untargeted data-dependent acquisition (DDA) analyses measured peptide size distribution, while amino acid analysis assessed nutritional content. The analysis of ten commercial CHs revealed similar amino acid profiles but varied peptide lengths, indicating diverse hydrolysis conditions. Products with higher proportions of smaller peptides showed elevated levels of the targeted bioactive peptides, suggesting that a smaller peptide size may increase bioactivity. These findings can inform the optimization of CH supplements, providing consumers with detailed peptide content for more informed choices. Data are available via ProteomeXchange with the identifier PXD051699.

## 1. Introduction

Collagen is an essential structural protein found abundantly in the animal kingdom, particularly as a major component of connective tissue in mammals. Known for its unique triple helix structure comprising the Gly-X-Y amino acid sequence, it contributes significantly to the mechanical properties of tissues [1,2,3]. The diverse types of collagen found in various tissues, such as type I in bone and type II in cartilage, highlight its physiological importance [4]. Collagen hydrolysates (CHs), derived from collagen through controlled enzymatic hydrolysis, generate low-molecular-weight peptides that are widely utilized for their bioactive potential in different industries, spanning health supplements to cosmetics [2,4,5].

As nutraceuticals, CHs have been investigated for their therapeutic value, including their ability to decrease joint pain associated with osteoarthritis, enhance skin health, and provide various other health benefits [4,5,6]. These hydrolysates contain bioactive dipeptides (DPs) and tripeptides (TPs), short chains of two or three amino acid residues, that exhibit significant biological functions and are increasingly studied as potential therapeutics in various diseases [7,8,9,10,11,12,13]. DPs and TPS are not only recognized for their contribution to energy production and nutritional regulation but also play a part in cell proliferation, inflammation, and nerve regulation [9,10,11], making them promising targets in medical research. Among these bioactive peptides found within CHs, three peptides stand out due to their notable physiological and biochemical properties: Hydroxyprolyl-glycine (Hyp-Gly), Glycyl-prolyl-hydroxyproline (Gly-Pro-Hyp), and Prolyl-hydroxyproline (Pro-Hyp). Hyp-Gly is acknowledged for its influence on collagen synthesis and potential benefits to skin health and tissue repair. Its presence serves as a building block for larger peptides and proteins essential for maintaining the structural integrity of the extracellular matrix [14,15,16]. Gly-Pro-Hyp, found in higher concentrations in some CH products, is not only pivotal for skin hydration and texture but has also been indicated to have an inhibitory effect on dipeptidyl peptidase-IV (DPP-IV), thus presenting potential therapeutic effects in the management of diabetes [17,18]. Pro-Hyp has garnered particular interest for its implication in maintaining collagenous tissue integrity and function, with studies suggesting that it may contribute substantially to the therapeutic effects perceived after the ingestion of CHs, such as decreased joint pain and amelioration of osteoarthritis symptoms [14,16,19,20].

The specificity and bioavailability of these DPs and TPs can, however, vary significantly depending on the source and processing of the CHs [3,4]. These variances presumably underlie the differences in peptide content and sequence, which affect their bioactivity. Elucidating this composition is, therefore, crucial both for the development of tailored nutraceuticals as well as the rigorous scientific understanding of their mechanisms of action. However, measuring DPs and TPs, especially within complex biological matrices, is technically challenging due to matrix interferences and potential signal suppression in mass spectrometry methods [12,21]. Liquid chromatography coupled with mass spectrometry (LC-MS) has become an important technique in DP quantification. For instance, Ozawa et al. [22] successfully employed both LC-MS and capillary electrophoresis–mass spectrometry (CE-MS) systems to quantify 335 distinct DPs, showcasing the versatility of these techniques. Similarly, Wu et al. [21], taking advantage of chemical derivatization using Dansyl chloride (Dns-Cl), quantified 361 DPs in complex samples, demonstrating LC-MS’s ability to provide rich qualitative and quantitative information. Another advanced method involved a pseudo-targeted liquid chromatography–tandem mass spectrometry (LC-MS/MS) approach for quantifying a comprehensive set of 400 DPs and 20 amino acids (AAs), combining Dns-Cl derivatization with a quantitative structure–retention relationship (QSRR) model, increasing the technique’s robustness and usefulness even without commercial standards for each analyte [23].

In this study, an advanced LC-MS/MS method was developed and thoroughly validated that incorporates propyl chloroformate (PCF) derivatization, allowing the quantification of three specific collagen-derived bioactive peptides in CHs: Hyp-Gly, Gly-Pro-Hyp, and Pro-Hyp. It is hypothesized that the application of the developed LC-MS/MS method will lead to the precise quantification of Hyp-Gly, Gly-Pro-Hyp, and Pro-Hyp levels in commercially sourced collagen hydrolysates (CHs). With the introduction of these novel analytical capabilities, the present study also aims to characterize the amino acid content, molecular weight distribution, and specific Hyp-Gly, Gly-Pro-Hyp, and Pro-Hyp content within ten commercially sourced CHs, leveraging the specificity and sensitivity of the novel LC-MS/MS methodology. By achieving this, it is hoped that the study will establish a foundational understanding of the quality and the potential therapeutic properties of these hydrolysates. This could have important implications for their application in health-related industries and for consumers who are seeking natural supplementation for various health issues.

## 2. Results

### 2.1. Quantification of Bioactive Peptides in Collagen Hydrolysates by Targeted LC-MS/MS

**Figure 1:**
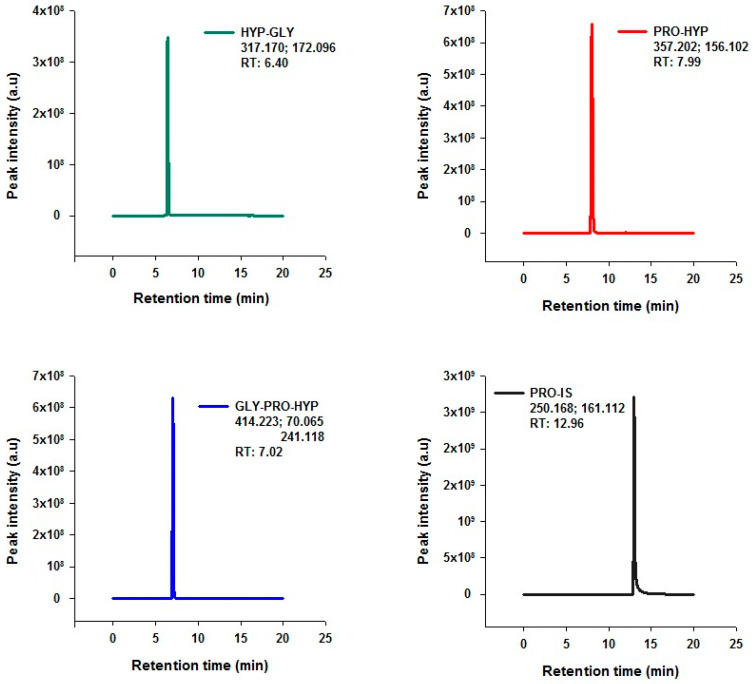
Extracted Ion Chromatogram (EIC) of Hyp-Gly, Pro-Hyp, Gly-Pro-Hyp, Internal standard Pro-IS. The numbers listed under the names of peptides represent the precursor ion *m/z* and product ion *m/z*. RT indicates the retention time.

A targeted LC-MS/MS methodology was developed for the measurement of di- and tripeptides from complex matrices constituting larger peptides, such as collagen hydrolysates. This method involved the derivatization of the peptides using PCF, enhancing the detection sensitivity and specificity of peptides against the complex background of collagen hydrolysates. Traditionally applied to amino acid analysis in GC and LC-MS setups, the adaptability of PCF derivatization to peptide mixtures has been demonstrated. Our pivotal observation of an interfacial precipitate layer—a phenomenon not seen during amino acid-only derivatization—suggests selective precipitation of larger peptides (Supplementary Figure S1). This layering effect facilitated by PCF suggests a dual role of improving the detection of smaller peptides and helping separate the larger ones, simplifying the sample for analysis. Subsequently, the derivatized peptide samples were analyzed using targeted LC-MS/MS analysis. The targeted LC-MS/MS analysis, conducted via Parallel Reaction Monitoring (PRM), was performed for the specific and sensitive quantification of the di- and tripeptides. In the analytical setup, the proline isotope standard (Pro-IS) was chosen as an internal standard due to its structural and chemical resemblance to the target dipeptides, ensuring consistent behavior during chromatographic separation and subsequent mass spectrometric detection. The inherent absence of Pro-IS in biological matrices further enhanced the accuracy of our quantitation by negating potential analytical interferences. Figure 1 illustrates the Extracted Ion Chromatogram (EIC) for each analyte, (a) Hyp-Gly, (b) Pro-Hyp, (c) Gly-Pro-Hyp, and (d) the internal standard Pro-IS (proline-containing internal standard). The chromatograms provide evidence of the method’s performance, showing clear peak resolution and the absence of interferences at the retention times of the target peptides and the internal standard.

The quantification of the peptides was facilitated using an internal standard calibration methodology, which employed a six-point linear calibration curve. This curve was constructed by analyzing mixtures of known concentrations of the dipeptides alongside a constant concentration of Pro-IS across calibration points. As a result, the response ratio of the analyte to the internal standard enabled the correction of any fluctuations in the instrumental parameters or sample-handling inconsistencies. The developed LC-MS/MS method demonstrated high specificity and sensitivity for the analysis of Hyp-Gly, Pro-Hyp, and Gly-Pro-Hyp in commercial collagen hydrolysates. The calibration curves exhibited excellent linearity for all three peptides, with correlation coefficients of 0.992, 1.000, and 0.995, respectively (Table 1). Limits of detection (LODs) and quantification (LOQ) were calculated for each peptide, with an especially low LOD observed for Pro-Hyp at 0.14 µM, indicating the method’s capacity for detecting even trace amounts of bioactive peptides (Table 1).

The quantitative analysis shows that the recovery percentages for all target peptides were within the acceptable range, with Hyp-Gly at 101.7%, Pro-Hyp at 100.5%, and Gly-Pro-Hyp at 99.9% at the lower QC level (Table 2). Precision, measured as the coefficient of variation (%CV), was under 5.5% for all peptides, demonstrating the method’s robustness and intra-day and inter-day repeatability.

**Figure 2:**
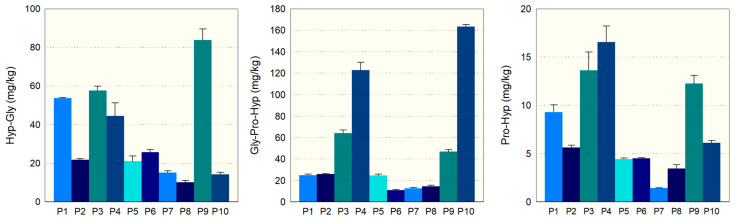
Bioactive peptide content of commercial CHs: Hyp-Gly, Gly-Pro-Hyp, Pro-Hyp.

To assess the broader analytical precision, an uncertainty budget was constructed factoring in sources of variability such as the calibration curve, intra-day and inter-day reproducibility, and the standards’ purity (Supplementary Table S1). Data were generated over three days, with four repeat measurements each day. The combined standard uncertainties for the respective compounds Gly-Pro-Hyp, Hyp-Gly, and Pro-Hyp were calculated to be 4.8%, 1.7%, and 6.2%. The targeted analysis of Hyp-Gly, Gly-Pro-Hyp, and Pro-Hyp dipeptides in commercial collagen hydrolysates (CHs) was conducted to evaluate their bioactive content. The developed LC-MS/MS method provided the quantification of dipeptide variations among ten CH products (P1 to P10). Figure 2 illustrates the bioactive content of ten different commercial CHs.

Hyp-Gly levels ranged from 10 mg/kg in P8 to 84 mg/kg in P9, indicating significant variations in dipeptide content among CH products. The relative standard deviation (RSD) was low for most products, suggesting consistent measurement accuracy. Gly-Pro-Hyp concentration showed considerable differences among the CHs, with P10 having an exceptionally high mean of 163 mg/kg compared to only 11 mg/kg in P6. The RSD values were generally low, indicating precise measurements within the batch of each product analyzed. The amount of the bioactive peptide Pro-Hyp also varied distinctly among the CH products, with P4 containing the highest content at 17 mg/kg and P7 the lowest at 1 mg/kg. Overall, the RSDs suggest good precision of the quantification process across the products tested.

### 2.2. Amino Acid Analysis

**Figure 3:**
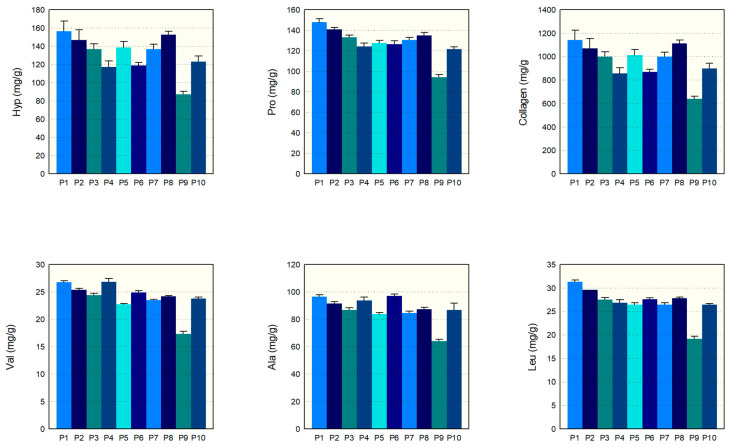
Amino acid and collagen content of commercial CHs.

The amino acid composition of ten commercial collagen hydrolysate products was examined, P1–P10, using validated analytical methods [24]. Figure 3 distinctly shows the levels of various amino acids and collagen content in these products. Our findings indicate that most collagen hydrolysates have similar amounts of key amino acids, like hydroxyproline, proline, valine, leucine, and alanine, except for P9, which initially had lower measurements. For instance, the hydroxyproline levels in P1 to P8 and P10 fell between 123.1 and 156.4 mg/g, and the proline levels fell between 121.7 and 147.7 mg/g. Similarly, the valine and leucine levels for these products were tightly packed between 23.6 and 31.1 mg/g, and 23.6 and 26.6 mg/g, respectively. The alanine content was also consistent, ranging from 83.5 to 97.0 mg/g in these samples. However, P9’s levels for these amino acids were notably lower before adjusting for the serving size. Collagen, the principal component we were investigating, showed a homogeneous content from 857.1 to 1142.8 mg/g in all samples except for P9, which had a value of 638.6 mg/g. However, when adjusting for P9’s larger serving size of 13 g compared to the 10 g for the other samples, its amino acid and collagen content fell in line with the rest. Overall, the analysis points to a consistent amino acid composition and collagen content among the CH samples tested. Additionally, the relative standard deviations (RSDs) range from 0.00% to 7.64%, further supporting the consistency of our measurements. The RSD ranges for each component are as follows: Hyp (2.36–7.64%), Pro (1.35–2.58%), Val (0.85–3.53%), Leu (0.00–3.16%), Ala (1.32–5.66%), and Collagen (2.35–7.62%). These RSD ranges illustrate that the variations in our measurements are minimal, thus supporting the consistency and reliability of our results.

### 2.3. Untargeted LC-MS/MS Analysis (DDA)

**Figure 4:**
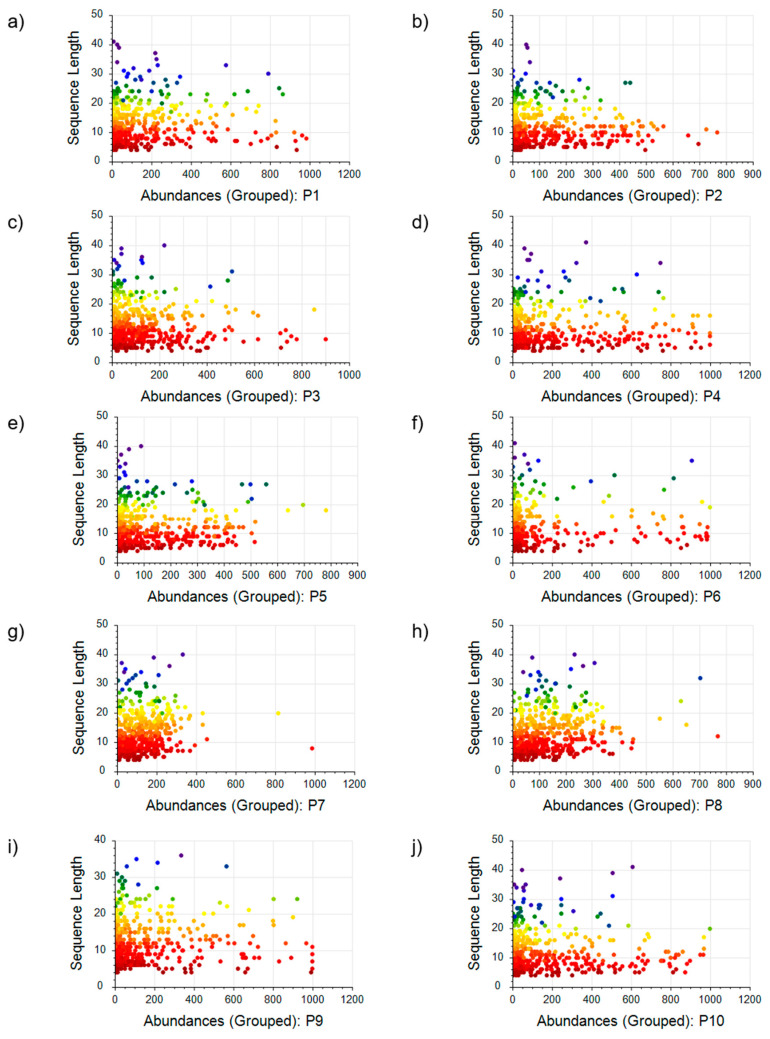
Scatter plot correlating sequence length with peptide abundance for peptides: (**a**) P1, (**b**) P2, (**c**) P3, (**d**) P4, (**e**) P5, (**f**) P6, (**g**) P7, (**h**) P8, (**i**) P9, (**j**) P10.

DDA MS was employed to analyze ten commercial collagen hydrolysate products, revealing a diversity of peptides in each. Proteome Discoverer 2.4 software, with its LFQ module, processed the raw spectral data. It was configured to identify peptides that were at least four amino acids long, which is the minimum length capable of being analyzed by the software. The peptide profiles were determined by their sequence and molecular weight distributions. The mass distribution of peptides P1–P10 from ten commercial collagen hydrolysates is visually represented in Figure 4 via scatter plots correlating sequence length with peptide abundance. The scatter plots reveal distinct patterns correlating the peptides’ physical dimensions with their prevalence, underlining the diverse qualitative and quantitative peptide makeup in the analyzed collagen hydrolysate product. The resulting peptide profiles, characterized by their individual sequences and molecular weight distributions, reveal distinctive signatures for each CH product. Table 3 summarizes the average abundance percentages of small, medium, and large peptides across the ten commercial collagen hydrolysate products.

In the investigation of peptide content in ten commercial collagen hydrolysate products, significant variability was observed in both peptide abundance and size distribution. The data were categorized by peptide sequence length into three groups: small peptides (4–12 amino acids), medium peptides (12–25 amino acids), and large peptides (25–41 amino acids). The analysis of average abundance reveals that small peptides were predominant in nearly all products. Specifically, products P1 and P5 showed small peptides constituting 52% and 66% of the peptide content, respectively. Remarkably, products P3 and P4 had a higher prevalence of small peptides, representing 74% and 76%. The most considerable rate of small peptides was detected in product P10, where they comprised 79% of the total peptide population. Medium-sized peptides displayed moderate abundance levels, generally composing a smaller proportion compared to small peptides. Product P7 was exceptional, with medium peptides being the majority at 53% of its content, significantly diverging from the other samples. The smallest proportions of medium peptides were found in products P2, P3, and P4, at 23%, 21%, and 18%, respectively. Large peptides were the least abundant across all samples. Nonetheless, their presence was notable, with contributions of 8% in product P1 and reaching a peak of 9% in products P6 and P8. The minimal occurrence of large peptides was noted in products P9, P4, P5, and P10, comprising only 3%, 6%, 6%, and 6% of the total peptide content, respectively. Overall, these data reflect a distinct trend where smaller peptides dominate the peptide profile in collagen hydrolysate products. The observed proportions of small, medium, and large peptides suggest a notable distribution pattern that could be influenced by the varying hydrolysis processes or starting materials used in the manufacture of these commercial offerings.

## 3. Materials and Methods

### 3.1. Chemicals and Materials

The reagents used in the study included the following NIMJ CRMs for the natural amino acid standards or calibration standards: L-alanine (Ala, CRM 6011-a, 99.9 ± 0.2%), L-leucine (Leu, CRM 6012-a, 99.9 ± 0.2%), L-proline (Pro, CRM 6016-a, 99.9 ± 0.2%), L-valine (Val, CRM 6015-a, 99.8 ± 0.2%), and trans-4-Hydroxy-L-proline (TCI Europe > 99.0%). For the ID-LC/MS method, internal standards of isotopically labeled amino acids were obtained from Cambridge Isotope Laboratories. The internal standards used were as follows: Ala (13C3, 99%; 15N, 99%), Leu (13C6, 99%; 15N, 99%), Pro (13C5, 99%; 15N, 99%), and Val (13C5, 99%; 15N, 99%). The standard materials used were Gly-Pro-Hyp (from Life Tein, Franklin Township, NJ, USA) and Hyp-Gly and Pro-Hyp (from Bachem, Switzerland).

The study utilized a selection of commercial collagen hydrolysates to investigate bioactive peptides. The chosen products included the following: P1: Collagen Peptides (Vital Proteins, Nestlé Health Science); P2: Collagen Peptides Bovine (Nature’s Supreme); P3: Collagen Peptides Bovine (Tennen Nutrition); P4: Marine Collagen (Supra Proteins); P5: Collagen For You (Supra Proteins); P6: Marine Collagen (Vital Proteins, Nestlé Health Science); P7: The Collagen All Body (Day2Day, Orzaks): P8: Collagen Peptides (Kiperin); P9: Three Peptides Complex (Barbaris); and P10: Beauty Hydrolyzed Collagen (VeNatura). These commercial hydrolysates were specifically chosen to encompass a diverse range of collagen sources, incorporating both bovine and marine origins. The selection process considered product characteristics and source diversity, aiming to capture a comprehensive representation of the bioactive peptides present in different collagen hydrolysates.

### 3.2. Preparation of Collagen Hydrolysates

Two grams of collagen hydrolysates was dissolved in 20 mL of 0.1 M HCl to create a homogeneous solution with a concentration corresponding to 700 µM of collagen. This stock solution was then further diluted using 0.1 M of HCl with a dilution factor of 100 to prepare the intermediate stock solution. For the amino acid analysis, the intermediate stock solution was further diluted sevenfold, resulting in a working solution with a final concentration equivalent to 1 µM of collagen.

For untargeted data-dependent analysis, the stock solution was diluted tenfold, achieving an approximate concentration of 70 µM of collagen.

For targeted LC-MS/MS analysis, a stock solution with 700 µM of collagen was used. For targeted LC-MS/MS analysis, a stock solution was used.

### 3.3. Preparation of Standard Solutions, Calibration Curves, and Quality

Lyophilized white powders of Hyp-Gly, Gly-Pro-Hyp, and Pro-Hyp peptides, with a purity of over 95%, were used to prepare the stock solutions. These powders were dissolved in 0.1 M of HCl to a final concentration of 1.00 mg/mL. Aliquots at 200 µL each were made and saved at −20 °C in polypropylene vials (Low Binding Tubes, Eppendorf, Germany). Calibration standards and Quality Control (QC) samples were accurately weighed and prepared on the day they were to be used. The calibration standards for Hyp-Gly and Gly-Pro-Hyp ranged from 2.5 to 80 µM, with specific values at 2.5, 5, 10, 20, 40, and 80 µM. Pro-Hyp standards were made with similar dilutions, achieving concentrations of 1.25, 2.5, 5, 10, 20, and 40 µM. The QC samples for Gly-Pro-Hyp and Pro-Hyp were prepared at 5 and 20 µM and, additionally, Gly-Pro-Hyp at concentrations of 15 and 7.5 µM. The working solutions were prepared by spiking 100 µL of the standard solutions with an equal volume of a labeled internal standard solution of proline (13C5, 99%; 15N, 99%) at a concentration of 1000 µM. Similarly, 100 µL of the peptide stock solution, equivalent to 700 µM collagen, was spiked with the same volume of the internal standard solution.

### 3.4. Derivatization for LC-MS/MS

The same derivatization procedure was applied for both amino acid analysis (AAA) and targeted LC-MS/MS analysis of the bioactive peptides. Briefly, 200 µL of the samples was mixed with 200 µL of propanol–pyridine (7:1) reagent and vortexed. Then, they were mixed with 200 µL of isooctane–PCF (propyl chloroformate) (5:1) and incubated for 1 min. Subsequently, 100 µL of CHCl_3_: isooctane–PCF (24:16:1) reagent was added, vortexed, and incubated for another minute. Finally, 200 µL of 5% HCl was added, and the mixture was vortexed and centrifuged. The upper phase of each sample was separated and transferred to clean tubes. The samples were then evaporated using a Caliper TurboVap LV evaporator under nitrogen. Lastly, each sample and the calibrants in the tubes were redissolved with 50 µL of buffer A of the corresponding method and transferred to suitable vials for LC-MS analysis.

### 3.5. Amino Acid Analysis (AAA)

The natural amino acid mixture was prepared gravimetrically in a ratio that closely matched the concentration of each amino acid in 1 µM of collagen. All these preparations were made fresh and independently for each hydrolysis reaction. The amino acid calibration standards was gravimetrically prepared, encompassing Valine (Val) at concentrations ranging from 10 to 350 µM, leucine (Leu) from 7.5 to 250 µM, alanine (Ala) from 10 to 300 µM, proline (Pro) from 7.5 to 200 µM, and hydroxyproline (Hyp) from 25 to 800 µM. Additionally, isotopically labeled amino acid solutions were prepared as a mixture, with each contributing a final concentration of 50 µM for Ala, Leu, Pro, and Val. Subsequently, a six-point calibration curve was gravimetrically established for each hydrolysis method by combining the natural and isotopically labeled amino acid mixtures in a one-to-one ratio. Six calibration standards were prepared, consisting of a mixture of natural and isotopically labeled amino acid solutions. Additionally, ten different collagen hydrolysates were prepared, each containing isotopically labeled amino acids. Following the weighing of solutions in glass tubes, they were transferred into separate Pico-Tag vials. Subsequently, both the calibration and sample mixtures were thoroughly dried under vacuum conditions using a Pico-Tag Workstation (Waters, Milford, MA, USA). The dried mixtures were then subjected to hydrolysis under inert atmosphere conditions at 130 °C for 24 h. This process involved using hydrolysis tubes containing 200 µL of 6 M constant boiling HCl and 1% phenol. Upon the completion of hydrolysis, the samples were evaporated under vacuum until completely dry. Finally, they were reconstituted with 200 µL of 0.1 M HCl in preparation for the amino acid derivatization process.

### 3.6. LC-MS Analysis

The amino acid analysis, untargeted DDA (data-dependent acquisition) analysis, and targeted LC-MS/MS analysis were conducted using a Dionex UPLC™ system (Thermo Scientific, Bremen, Germany) coupled with a Q Exactive HF-X Hybrid Quadrupole–Orbitrap Mass Spectrometer (Thermo Scientific, Bremen, Germany), equipped with an electrospray ionization source.

#### 3.6.1. Targeted LC-MS/MS Analysis of Bioactive Peptides

Chromatographic separation was performed using an Aeris Peptide C18 column (250 × 2 mm i.d., with a 5 µm particle size). The mobile phase consisted of two components: Mobile phase A was 95% water and 5% acetonitrile with 0.1% formic acid; and mobile phase B was 95% acetonitrile with 5% water, also including 0.1% formic acid. The gradient began at 10% of solvent B, increasing to 30% in the first 3 min, and reaching 50% within 10 min, and was followed by appropriate washing and re-equilibrating with solvent B. The flow rate was set at 0.35 mL/min, and the column temperature was maintained at 40 °C. An injection volume of 5 µL was used for each sample. To minimize contamination of the MS system, a divert valve was utilized with positions 1–6 denoting the waste path and 1–2 directed to the MS. The divert valve was scheduled as follows: from 0 to 6 min and 8.5 to 12 min to waste (1–6); from 6 to 8.5 min and 12 to 14 min to the MS (1–2); and from 14 to 22 min back to waste (1–6).

The instrument settings were optimized for each peptide through direct injection. The ESI source spray parameters included a source voltage of 3.5 kV, a sheath gas setting of 48, a capillary temperature of 320 °C, an S lens RF level of 50, and an auxiliary gas heater at 190 °C. Parallel Reaction Monitoring (PRM) transitions were employed for sensitive detection, as detailed in Table 4.

The MS and MS/MS parameters were as follows: The full MS scans were run with a resolution of 120,000. An AGC target of 3 × 10^6^ and a maximum IT of 200 ms were set, with a full MS scan range from a 133 to 2000 mass-to-charge ratio (*m/z*). For MS/MS in PRM mode, a resolution of 30,000 was chosen. The AGC target for MS/MS was 2 × 10^5^, with a max IT of 100 ms. The system executed 4 loop counts, targeting the top 4 ion intensities for fragmentation. An isolation window of 4.0 *m/z* was set for ion selection. The quantitation approach was ID/MSMS with a six-point linear calibration curve for accurate measurements. ThermoXcalibur 4.1.31.9 Quan Browser software was used for quantification. “The mass spectrometry proteomics data have been deposited to the ProteomeXchange Consortium via the PRIDE (Vizcaíno et al., 2015) [25,26] partner repository with the dataset identifier PXD051699”.

#### 3.6.2. Amino Acid Analysis

Amino acid analysis (AAA) was performed based on the method described in detail in the article by Oztug et al., 2024 [24]. An additional amino acid, Hyp, was included in the analysis. The detected *m/z* value for Hyp after PCF derivatization is 260.148. Proline IS (internal standard) was used for Hyp quantification.

#### 3.6.3. Untargeted LC-MS/MS Analysis (DDA)

Peptide separation was conducted using reversed-phase liquid chromatography (RP-LC) on an Aeris Peptide C18 column (250 × 2 mm i.d., 5 µm particle size). The mobile phases comprised two solutions: Mobile phase A consisted of 95% water (H_2_O) and 5% acetonitrile, with 0.1% formic acid (FA), and mobile phase B was made up of 95% acetonitrile and 5% water, also containing 0.1% FA. The analysis was performed with a constant flow rate of 350 µL/min. The gradient program started with 0% of solvent B, increased to 5% over the first 7 min, and reached 50% in 50 min, followed by subsequent wash and equilibration steps. The column temperature was maintained at 30 °C throughout the analysis, while the injection volume for each sample was 10 µL. Samples were prepared at a final concentration of 9 mg/mL in a solvent composed of 95% water. The instrumental settings were as follows: the spray voltage was set to 3.5 kV, the funnel RF level to 50, and the capillary temperature to 320 °C. The device was set to operate in a “Full MS/DD-MS/MS” mode for DDA analysis. The Full MS resolution was set at 120,000 for *m/z* 200, with a Full MS AGC (Automatic Gain Control) target of 3 × 10^6^ at a maximum injection time of 45 ms. The defined mass range spanned from 150 to 2250. The MS/MS setup included an AGC target of 1 × 10^5^, a resolution of 15,000, a maximum injection time (max IT) of 22 ms, an intensity threshold of 2 × 10^4^, and an isolation width of 1.3 *m/z*. The normalized collision energy was fixed at 28%, and all data acquisition was performed in the positive ion mode. Raw data were searched against a fasta file containing collagen sequences from multiple species, including bovine and salmon, using the Sequest HT search engine [27]. A false discovery rate (FDR) of 0.01 was applied. The Sequest HT settings included a precursor mass tolerance of 10 ppm and fragment mass tolerance of 0.02 Da. Variable modifications included oxidation and N-terminal acetylation. Proteins were identified with at least two peptides of a minimum of four amino acids in length. Protein quantification was performed using label-free quantification (LFQ) in Proteome Discoverer 2.4. Furthermore, the distribution of each peptide was visualized by creating histograms of each peptide based on the theoretical monoisotopic mass (MH+) in Dalton (Da) units with the help of Proteome Discoverer 2.4.

## 4. Discussion

The targeted LC-MS/MS method developed in this study, incorporating PCF derivatization, effectively validated the initial hypothesis. This technique demonstrates high sensitivity and is capable of measuring specific bioactive dipeptides and tripeptides in the complex matrix of collagen hydrolysates (CHs). The advantage of including PCF derivatization is that it increases the sensitivity of DP and TP detection, creating derivatives that ionize more readily, leading to lower quantification limits. Moreover, during sample preparation, PCF derivatization induces the precipitation of larger peptides, simplifying the matrix and minimizing potential interferences during mass spectrometric analysis. The greatest advantage of the method is that no pre-separation techniques were employed to separate di- and tripeptides from large peptides and proteins. While Xu et al. [21] utilized the methanol chloroform extraction method for metabolite separation, Ozawa et al. [22] employed a 5 kDa cutoff filter, and Tang et al. [23] precipitated proteins with methanol. All these separation methods introduce additional sources of variation. This study marks the first measurement of dipeptides in a peptide solution with complete validation of the method’s criteria. Moreover, the coefficient of variation (CV) is below 15%, which is lower compared to previous studies [22,23]. The method’s high precision and reproducibility allow for the accurate quantification of Hyp-Gly, Gly-Pro-Hyp, and Pro-Hyp and reveal subtle variations in their levels across different commercial CHs. Commercial CH products typically lack detailed information regarding their bioactive peptide content. While labeling usually focuses on amino acid and overall collagen quantities expressed in mg/g, this study brings to light the crucial importance of specifying bioactive dipeptide and tripeptide contents. Notably, even products marketed as having high bioactivity often do not provide data on these specific peptides, highlighting the novelty of this research. For the first time, the levels of Hyp-Gly, Gly-Hyp, and Gly-Hyp-Pro in commercially available hydrolysates have been quantified, paving the way to tailor serving sizes based on cellular and clinical dose-dependent studies, enhancing the efficacy of CH supplements and informing consumer choice.

The peptide size distribution within CHs shows considerable variability. Without explicit knowledge of the hydrolysis methods used for these proprietary commercial products, a direct commentary on the relationship between processing techniques and peptide size distribution cannot be made. However, the measurement methodology established allows for the assessment and comparison of different hydrolysis methods by mapping out the bioactive peptide content using the approach presented here, which could lead to the determination of optimal conditions for hydrolysis, ensuring the efficient generation of small and bioactive peptides. The DDA analysis, despite its limitation of identifying peptides of at least four amino acids, provided essential insights into the peptide composition of commercial collagen hydrolysates. The smaller peptides displayed a higher average abundance, indicative of efficient hydrolysis in breaking down the native triple helical structure of collagen into smaller, more bioavailable molecules. The relative abundance of small peptides in products like P3, P4, and notably P10—which constituted 79% of its peptide content—highlights these hydrolysates’ potential to deliver bioactive compounds. Reinforcing this observation, the targeted LC-MS/MS analysis reveals that P3 and P4 have higher levels of the bioactive peptides Hyp-Gly, Gly-Pro-Hyp, and Pro-Hyp, which are reputed for their therapeutic roles in human health. These peptides are associated with stimulating collagen production, inhibiting enzymes that degrade collagen peptides, and possibly reducing inflammation—benefits that are particularly sought after in nutraceuticals and functional foods. Furthermore, the targeted analysis highlights that P10 has the highest concentration of Pro-Hyp, a tripeptide particularly recognized for its effects on skin and joint health. This finding aligns with the DDA results, which show a higher proportion of small peptides in P10, indicating consistency between the methods and implicating a strong presence of bioactive compounds. This congruence between the peptide size distribution from the DDA analysis and the specific quantification of bioactive peptides through targeted LC-MS/MS underscores a critical point: the smaller the peptides in CH products, the higher the likelihood of enhanced bioactive potential. The parallel results from both analytical approaches validate the reliability of the methods used. Conversely, the significant presence of medium-sized peptides in product P7, accounting for more than half of its peptide content, raises questions about the specific hydrolysis conditions employed. The lesser abundance of large peptides across all products aligns with the anticipated outcome of enzymatic hydrolysis, which is intended to reduce the size of the protein for enhanced digestibility and bioavailability. Still, the consistency in their minor presence suggests a controlled process that limits the extent of hydrolysis, preventing the complete degradation of collagen into its amino acid components.

The uniform amino acid profiles identified in our analysis of commercial collagen hydrolysate products indicate that the industry predominantly uses standardized starting materials and quantities of collagen. Despite this homogeneity in amino acids, the diversity in peptide size distribution among the products points to distinct hydrolysis conditions. It is these variations in enzymatic processing that are likely responsible for the different bioactive peptide profiles observed, which ultimately could influence the health benefits attributed to each collagen hydrolysate supplement.

## 5. Conclusions

In summary, in this study, a novel LC-MS/MS method utilizing PCF derivatization was developed, enabling the precise measurement of specific dipeptides and tripeptides in collagen hydrolysates. The implications of our findings extend into practical applications, where the identification of small and bioactive peptides in CH products can guide optimization processes in manufacturing. Enzymatic hydrolysis conditions can be tailored to yield collagen hydrolysates with specific bioactive profiles suited to targeted health outcomes. Producers may adjust their processes to increase the yield of certain bioactive peptides, thereby enhancing the therapeutic potential of their products and aiding consumers in making informed choices about their CH product selections based on their health goals. More data are needed for the identification and quantification of key bioactive peptides to provide a better understanding of the potential therapeutic properties and qualities of these products. Future studies should expand the sample size, include a broader range of products, and utilize different hydrolysis conditions to validate and enhance the findings presented here.

## Figures and Tables

**Table 1:** Validation parameters of targeted LC-MS/MS method for Hyp-Gly, Pro-Hyp, and Gly-Pro-Hyp peptides.

Parameters	Hyp-Gly	Pro-Hyp	Gly-Pro-Hyp
Linearity (µM)	0–80	0–40	0–80
Correlation coefficient	0.992	1.000	0.995
Limit of detection, LOD (µM)	1.40	0.14	1.16
Limit of quantification, LOQ (µM)	4.19	0.43	3.38

**Table 2:** % Recovery and quantitative results.

Peptide		Expected Concentration (µM)	Measured Concentration (*n* = 4) (µM)	Precision (%CV)	Recovery (%)
**Hyp-Gly**	QC-1	7.4	7.5	5.5	101.7
	QC-2	15.7	15.3	1.8	97.1
**Pro-Hyp**	QC-1	4.7	4.7	1.0	100.5
	QC-2	20.1	21.1	0.8	105.0
**Gly-Pro-Hyp**	QC-1	4.5	4.4	1.5	98.0
	QC-2	19.0	19.0	0.3	99.9

**Table 3:** Peptide size distribution profile in commercial collagen hydrolysates.

	P1	P2	P3	P4	P5	P6	P7	P8	P9	P10
Small peptides (Seq length 4–12) (Avg. abundance) %	51	73	74	76	66	65	42	58	68	80
Medium peptides (Seq length 12–25) (Avg. abundance) %	41	23	21	18	28	26	53	33	29	14
Large peptides (Seq length 25–41) (Avg. abundance) %	8	4	5	6	6	9	5	9	3	6

**Table 4:** Ions/PRM transitions monitored.

Peptide Name	Precursor Ion	Product Ion 2+	Collision Energy (NCE)
Pro (13C5, 99%; 15N, 99%)	250.168	161.112	30
Hyp-Gly	317.170	172.096	40
Gly-Pro-Hyp	414.223	70.065; 241.118	28
Pro-Hyp	357.202	156.102	40

## Data Availability

Data are available via ProteomeXchange with the identifier PXD051699.

## Supplementary Materials

The following supporting information can be downloaded at: https://www.mdpi.com/article/10.3390/molecules29112592/s1, Figure S1: PCF Derivatization mixture of collagen hydrolysates; Table S1: Uncertainty budget for quantification of di and tri peptides in CHs using targeted LC-MS/MS analysis; Figure S2: Mass Distribution Histograms Based on Theoretical Monoisotopic Mass (MH+) in Dalton (Da) for Peptides

## Abbreviations

AAA	Amino acid analysis
CE-MS	Capillary electrophoresis–mass spectrometry
CH	Collagen hydrolysate
DDA	Data-dependent acquisition
Dns-Cl	Dansyl chloride
DP	Dipeptide
TP	Tripeptide
DTT	Dithiothreitol
FASP	Filter-aided sample preparation
FDR	False detection rate
HYP	Hydroxyproline
LC-hrMS	Liquid chromatography–high-resolution mass spectrometry
LC-MS	Liquid chromatography–mass spectrometry
LC-MS/MS	Liquid chromatography–tandem mass spectrometry
LFQ	Label-free quantitation
PCF	Propyl chloroformate
PRM	Parallel Reaction Monitoring
RP-LC	Reversed-phase liquid chromatography
UPLC	Ultra-performance liquid chromatography
