# Supplementary material for: Bioactive Peptide Profiling in Collagen Hydrolysates: Comparative Analysis Using Targeted and Untargeted Liquid Chromatography–Tandem Mass Spectrometry Quantification
Source: Molecules. 2024 May 31;29(11):2592. doi: 10.3390/molecules29112592 (PMC11173644; doi:10.3390/molecules29112592)
Supplement: Supplementary file 1 [file molecules-29-02592-s001.zip › molecules-3027372-supplementary.pdf]

Bioactive Peptide Profiling in Collagen Hydrolysates: Comparative Analysis Using Targeted and Untargeted LC-MS/MS Quantification"

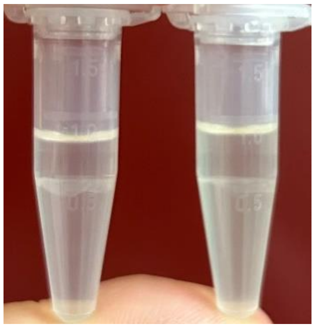

Supplementary Figure S1 PCF Derivatization mixture of collagen hydrolysates

Supplementary Table S1. Uncertainty budget for quantification of di and tri peptides in CHs using targeted LC-MS/MS analysis

	**Gly-Pro-Hyp**	**Hyp-Gly**	**Pro-Hyp**
µM	90.5	114.4	24.6
Uncertanity Components (Relative %)			
Inter-Day Variation (AA) (%)	4.1	0.3	1.4
Intra-Day Variation (AA) (%)	1.9	0.1	6.0
Calibration Curve (%)	1.8	1.6	1.0
Purity of amino acid std (%)	0.1	0.1	0.1
The accuracy of Balance (%)	0.0	0.0	0.0
Combined Standard Uncertanity (%)	4.8	1.7	6.2
Combined Standard Uncertanity (mg /kg)	4.4	1.9	1.5
Expanded Standard Uncertanity (k=2)	8.8	3.8	3.0

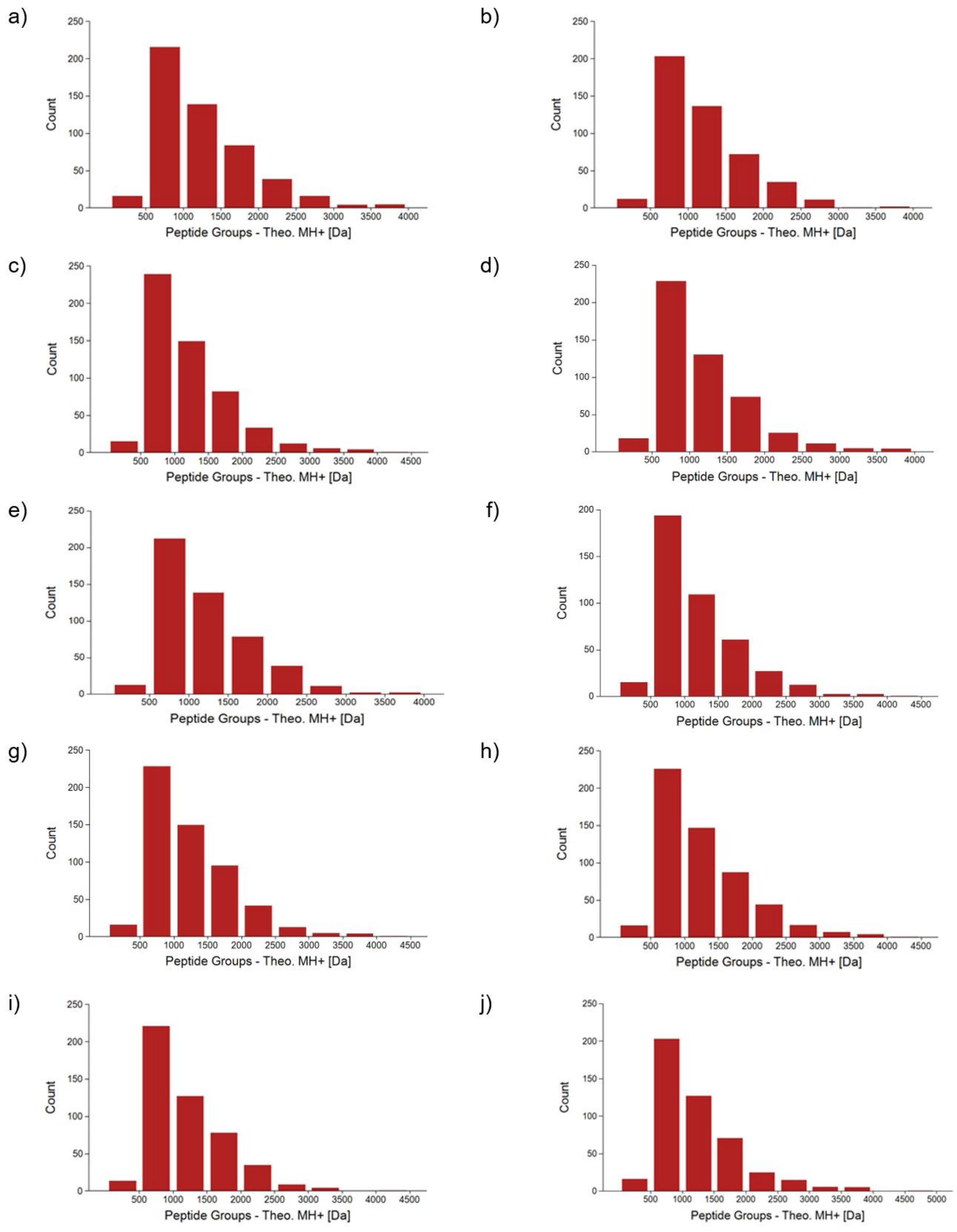

Supplementary Figure S2 Mass Distribution Histograms Based on Theoretical Monoisotopic Mass (MH+) in Dalton (Da) for Peptides (a) P1, (b) P2, ..., (j) P10 These histograms showcased the molecular weight profiles of each peptide, providing a clear depiction of the variations in peptide size characteristic of each sample.
